# Combining 3D printing technology with customized metal plates for the treatment of long segment femoral shaft comminuted fractures

**DOI:** 10.3389/fsurg.2026.1705131

**Published:** 2026-01-26

**Authors:** Rongda Xu, Yingying Liang, Hanfei Liu, Jiahui Li, Xueting Zhou, Ming Sun, Hongliang Tu, Zelin Zhang, Siyu Duan, Zhencun Cai

**Affiliations:** 1 Department of Orthopedic Surgery, The Second Affiliated Hospital of Shenyang Medical College, Shenyang, Liaoning, China; 2 Department of Orthopedic Surgery, Shengjing Hospital of China Medical University, Shenyang, Liaoning, China; 3 Department of Orthopedic Surgery, Central Hospital Affiliated to Shenyang Medical College, Shenyang, Liaoning, China; 4 Liaoning Province Key Laboratory for Phenomics of Human Ethnic Specificity and Critical Illness, Shenyang Key Laboratory for Phenomics, Shenyang Medical College, Shenyang, Liaoning, China

**Keywords:** 3D printing technology, comminuted, customized metal plates, femoral shaft fractures, long segment

## Abstract

**Objective:**

This study aims to evaluate the clinical value of combining 3D printing technology with customized metal plates in the treatment of long-segment femoral shaft comminuted fractures.

**Methods:**

A retrospective study was conducted on 36 patients with long-segment femoral shaft comminuted fractures who were treated at our hospital between September 2020 and September 2023. Patients were divided into two groups: the conventional group (18 patients), treated with limited open reduction and intramedullary nailing, and the 3D printing group (18 patients), which utilized 3D-printed models and customized metal plates for assisted internal fixation. Intraoperative evaluation metrics included surgical time, number of fluoroscopy exposures, and intraoperative blood loss. Postoperative evaluation metrics included the time to weight-bearing initiation, time to full weight-bearing, and fracture healing time. At 3 months post-operation and at the final follow-up, evaluations were conducted on the knee flexion-extension range of motion (ROM), hospital for special surgery (HSS) score, hip flexion-extension ROM, Harris score, visual analogue scale (VAS) score, and the occurrence of complications. At the final follow-up, lateral displacement, angular deformity, shortening deformity, and the modified radiographic union score for tibia (mRUST) score of the fracture site were evaluated.

**Results:**

The 3D printing group had significantly shorter surgical time and fewer fluoroscopy exposures (both *P* < 0.001), while intraoperative blood loss was higher but not statistically significant (*P* = 0.252). The 3D printing group also showed faster initiation of partial weight-bearing, full weight-bearing, and fracture healing (*P* < 0.001, *P* < 0.001, *P* = 0.009). At 3 months and final follow-up, the 3D printing group showed significantly better knee flexion-extension ROM, HSS score, hip flexion-extension ROM, and Harris score than the conventional group (all *P* < 0.001), while VAS scores showed no significant difference (all *P* > 0.05). At the final follow-up, the 3D printing group demonstrated better results in terms of lateral displacement, angulation deformity, shortening deformity, and mRUST score (all *P* < 0.001).

**Conclusion:**

Combining 3D printing technology with customized metal plates in treating long-segment femoral shaft comminuted fractures improves surgical efficiency, fracture reduction and healing quality, and promotes functional recovery.

## Introduction

A long-segment comminuted femoral shaft fracture refers to a severe fracture of the femoral shaft over a relatively large area, accompanied by the formation of multiple fracture fragments. It typically involves a longer segment of the bone and a higher degree of fragmentation, making it a complex type of fracture. For most femoral shaft fractures, intramedullary nailing is typically considered the preferred treatment option (1–3). Although intramedullary nailing provides reliable fracture fixation, plate fixation may offer more significant advantages in achieving anatomical reduction in certain specific types of fractures (4). In the treatment of comminuted femoral shaft fractures, significant displacement or rotation of fracture fragments after intramedullary nail implantation may result in fracture malalignment. At the same time, the instability of the intramedullary nail fixation may further affect the alignment of the fracture. This condition not only delays fracture healing but may also increase the risk of nonunion (5, 6). In this regard, although plate fixation may cause significant disruption to the surrounding soft tissues and bone blood supply, increasing the risk of delayed union or nonunion, it remains an effective and viable alternative for managing complex fractures.

However, traditional plate fixation still faces many challenges when dealing with fractures that involve a large length, numerous fragments, and complexity. Especially, conventional internal fixation plates are usually manufactured based on standardized sizes, often failing to meet the actual requirements due to insufficient length (7). In addition, conventional plates also have issues such as difficulty in reduction and insufficient fixation stability. To overcome these limitations, personalized fracture fixation devices have emerged as an important approach to improving treatment outcomes.

In recent years, 3D printing technology has been widely applied in the field of personalized medicine, particularly showing unique advantages in the treatment of complex fractures. In our previous research, we found that 3D printing technology offers significant benefits in the treatment of acetabular fractures and intra-articular fractures (8–10). The application of this technology in the treatment of complex fractures primarily lies in printing precise bone models to assist in diagnosis and surgical planning. These models not only help doctors make more accurate preoperative predictions and plans but also provide intuitive references during the surgery, thereby improving the precision and safety of the procedure (11, 12). Based on the above research findings, this study further expands the application of 3D printing technology, focusing on analyzing its clinical application effectiveness in the treatment of long-segment femoral shaft comminuted fractures.

Currently, research on the use of 3D printing technology combined with personalized plates for the treatment of long-segment comminuted femoral shaft fractures remains limited. Our hypothesis is that, compared to traditional treatment methods, the treatment plan combining 3D printing technology with personalized plates can provide more precise fracture reduction and stability, thereby improving the treatment outcomes for patients with long-segment femoral shaft comminuted fractures and promoting earlier functional recovery.

## Materials and methods

### Participants

A retrospective analysis was conducted on 36 patients with long-segment femoral shaft comminuted fractures treated in the Department of Orthopedic Surgery at the Second Affiliated Hospital of Shenyang Medical college from September 2020 to September 2023. The degree of comminution in all fractures was classified according to the Winquist-Hansen classification system (13). For the definition of long-segment fractures, we defined a long-segment fracture as one where the length of the fracture line exceeds 50% of the femoral shaft length. Inclusion criteria are as follows: (1) Age ≥18 years; (2) Fracture located from below the lesser trochanter to above the femoral condyle; (3) Comminuted fractures classified as Winquist-Hansen type II-IV; (4) Fracture line length exceeding 50% of the femoral shaft length; (5) Fresh closed fractures (occurring within the past 3 weeks); (6) Voluntarily accepted treatment with 3D printing technology combined with personalized plates and signed informed consent. Exclusion criteria include: (1) Open or pathological fractures; (2) Old fractures (fracture duration >3 weeks); (3) Comorbid with other types of fractures; (4) Pre-injury inability to move the lower limb due to other reasons; (5) Loss to follow-up or incomplete follow-up data; (6) Comorbid with other severe complications affecting postoperative recovery; (7) Received other treatments that may affect fracture healing, such as long-term steroid use.

Based on the treatment method, 18 patients who received treatment with 3D printing combined with customized metal plates were defined as the 3D printing group, while the other 18 patients who received limited open reduction and intramedullary nailing were defined as the conventional group. The general data, including age, gender, BMI, injury mechanism, and fracture classification, were recorded for both groups of patients. This study obtained approval from the Medical Ethics Committee of the Second Affiliated Hospital of Shenyang Medical college (Approval No.: 2020132). All patients participating in the study were informed and agreed that their personal information and clinical data would be used for research analysis. Before the start of the study, all patients signed a written informed consent form. The postoperative follow-up period for all patients was at least 12 months or until the fracture was fully healed.

### 3D model production

**Figure 1:**
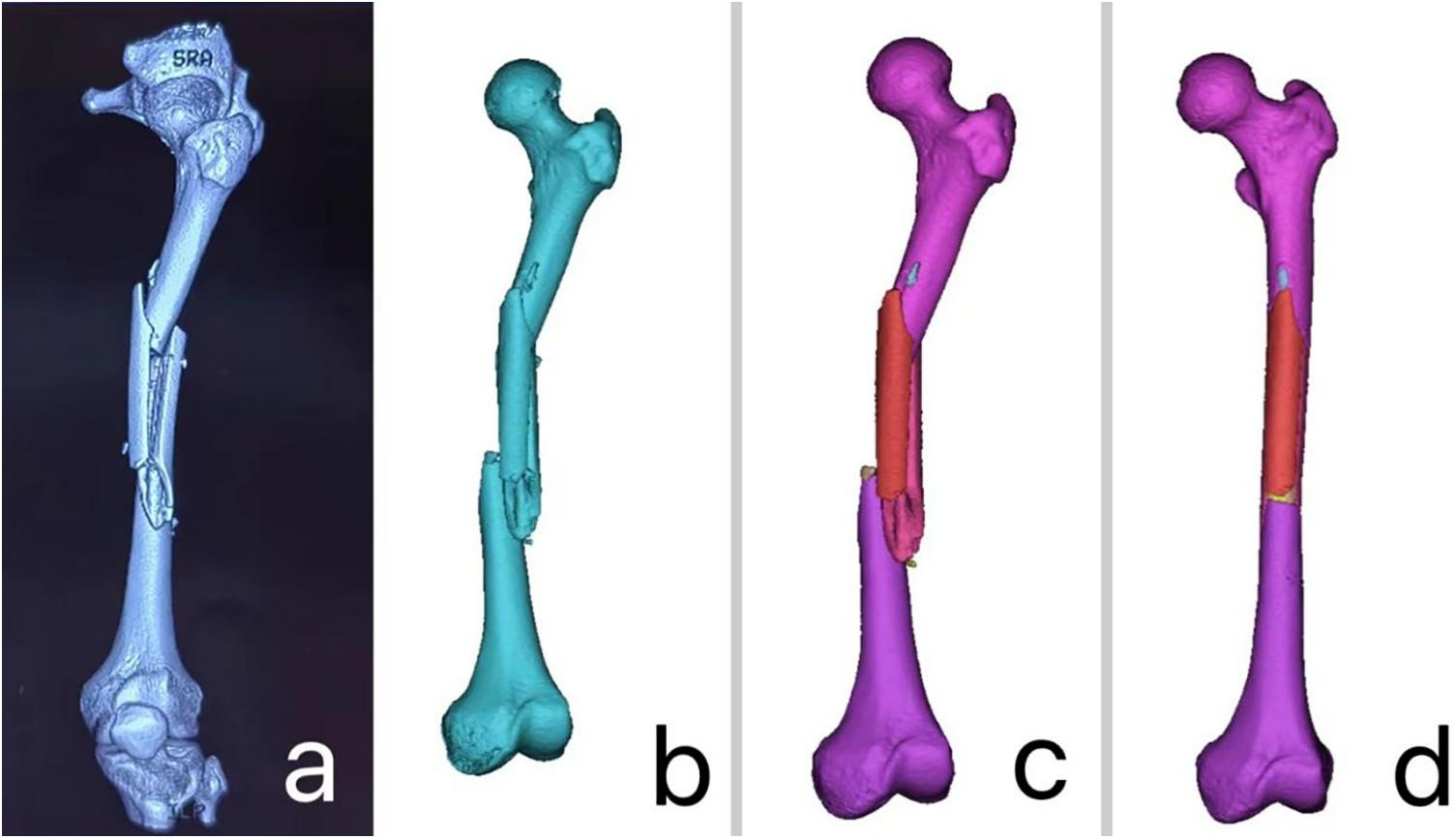
**(a)** CT data of the patient's fracture site; **(b)** 3D modeling of the fracture data using mimics software; **(c)** extracting each bone fragment using the region segmentation tool and color-coding them to display in different colors; **(d)** virtual anatomical reduction of the fracture fragments using the move and rotate functions.

**Figure 2:**
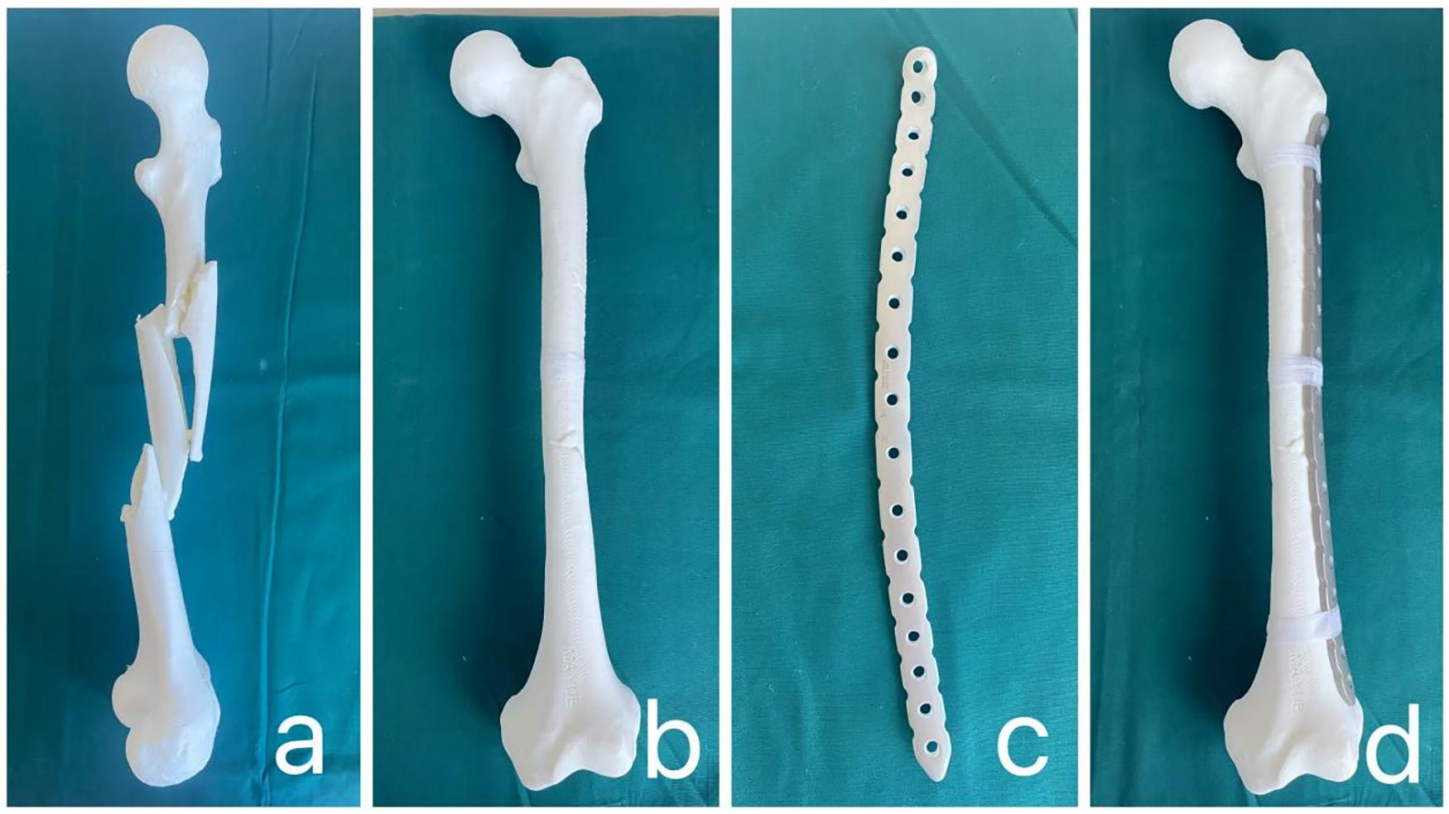
**(a)** 1:1 scale physical model of the fracture printed using a 3D printer; **(b)** 3D printed model created after virtual reduction; **(c)** physical image of the personalized plate; **(d)** Fit of the virtual reduced physical model and the personalized customized plate.

The CT data of the patient's fracture site (Figure 1a) were imported into Mimics 20.0 software (Materialise, Belgium) in DICOM format. In Mimics, advanced image processing functions were used for precise region segmentation and three-dimensional reconstruction of the CT images. By adjusting appropriate thresholds to fine-tune the resolution of soft tissue and bone tissue, a 3D model of the fracture site was successfully generated (Figure 1b). Further, the editing tools in Mimics were applied to remove noise and unnecessary details from the images. The region segmentation tool was used to extract each bone fragment, which was then color-coded to display in different colors (Figure 1c). Virtual anatomical reduction of the fracture fragments was performed using the move and rotate functions (Figure 1d). Next, the 3D fracture model and the data after virtual reduction were exported as STL files and imported into FashPrint 5 software (FlashForge, China). Polylactic Acid (PLA) was used as the printing material, with a typical printing temperature of 215–220 °C for standard PLA filaments. The converted files were transferred to a 3D printer (Dreamer, Dongya Medical, Shenyang, China), and parameters such as printing speed, layer thickness, and fill density were adjusted as needed. After printing, the models were properly cooled and removed from the mold, resulting in a 1:1 scale physical model of the fracture (Figure 2a) and the model after virtual reduction (Figure 2b).

### Designing a individualized metal plate

Using Unigraphics NX software (Siemens PLM Software, USA), the placement, shape, and size of the personalized plate were designed in detail based on the virtual reduction of the fracture. This process involved key factors such as the plate's placement, curvature, arrangement, number, and positioning of the screw holes. Subsequently, PLA was used as the printing material to print a physical model of the plate using FashPrint 5 software. Then, the physical model of the plate was imported into Mimics 20.0 software via reverse scanning, and the personalized plate was simulated for placement on the virtual fracture model to confirm its position. To further verify the design, we applied transparency processing to the fracture model, allowing clear observation of the screw positions and implantation angles. The measurement function was used to accurately confirm the screw lengths. Based on these data, special screw holes were created on the plate. Finally, pure titanium TA3 was used as the raw material, and the plate was processed at a manufacturing facility to produce a real personalized plate that met the design requirements (Figure 2c). During the preoperative simulation, we found that the personalized plate perfectly fit the reduced model (Figure 2d).

The personalized titanium alloy plate used in this study had a uniform thickness of 4.5 mm and a hole spacing of 20 mm. The mechanical performance of the plate was evaluated using static four-point bending and bending fatigue tests on an INSTRON 8801 servo-hydraulic testing system, while axial pull-out tests of the screws were performed using a universal testing machine to ensure the overall fixation strength and stability of the plate–screw construct.

### Preoperative preparation

After admission, a thorough routine examination was conducted for the patient. During the preoperative preparation phase, temporary stability was provided by tibial tuberosity traction to maintain the normal length of the limb. All patients undergo anteroposterior and lateral x-ray imaging of the femoral shaft before surgery, and CT scans and three-dimensional reconstruction techniques were used to evaluate the fracture in detail. In the 3D printing group, 3D models of the fracture site were printed based on the CT imaging data, and personalized plates were designed according to the anatomical characteristics of the patient.

### Surgical procedure

The patient was positioned appropriately and undergoes general anesthesia. Routine disinfection was performed using iodine tincture, and a sterile drape was applied. All surgical procedures were performed by a team of experienced surgeons working together.

Conventional group: After the anesthesia takes effect, the patient was positioned supine on a traction bed, and traction was applied to the affected limb to restore the femoral shaft length. A longitudinal incision was made at the proximal point of the greater trochanter, followed by sequential dissection of the skin, subcutaneous tissue, and fascia layers. The gluteus maximus muscle was bluntly separated. At the proximal point of the greater trochanter, the femoral shaft was opened and a guide pin was inserted. “Golden finger” (a reduction instrument) was used to assist in reduction. If closed reduction fails, a longitudinal incision was made at the lateral midshaft of the femur, and layers were opened sequentially while minimizing periosteal stripping. The fracture ends were cleaned, bone callus was removed, and the medullary cavity was cleared. Fracture reduction was performed under direct vision. To ensure stability, titanium cables were used to bind and fix the fracture ends. After confirming the fracture alignment and reduction were satisfactory under C-arm fluoroscopy, a guide pin was inserted at the apex of the greater trochanter. The intramedullary cavity was reamed along the guide pin, and an appropriately sized intramedullary nail was inserted. Once fluoroscopy confirms the correct length, diameter, and position of the intramedullary nail, two locking screws were placed in the distal femur using a guide. The intramedullary nail was tapped to compress the fracture ends. Subsequently, two more locking screws were inserted at the proximal femur using the guide. After confirming the fracture reduction and the proper position of the internal fixation under C-arm fluoroscopy, the tail cap of the intramedullary nail was tightened.

**Figure 3:**
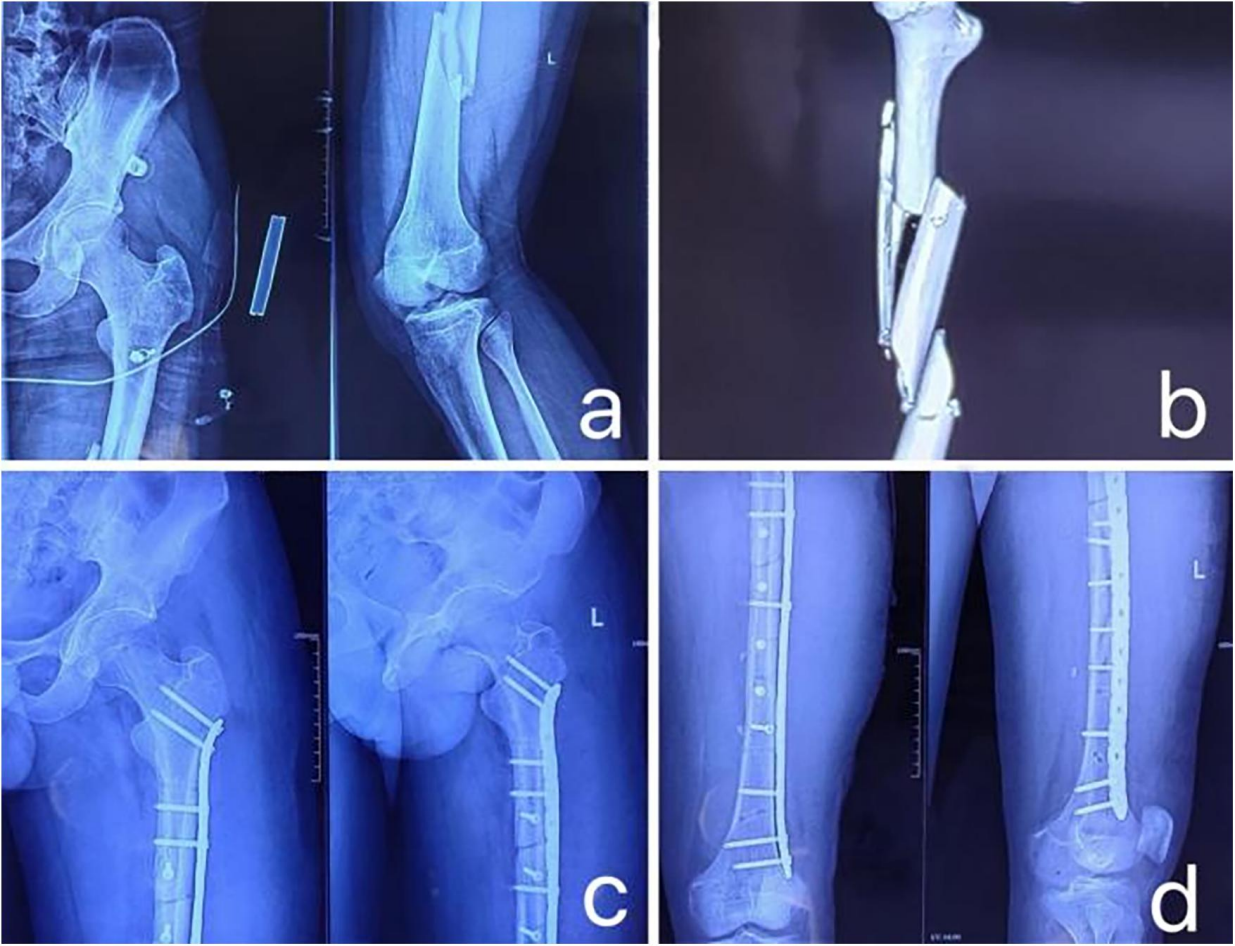
**(a)** x-ray image on the day of admission; **(b)** 3D CT scan performed on the day of admission, showing a long segment comminuted fracture of the midshaft of the femur; **(c,d)** x-ray images of the femoral shaft in anteroposterior and lateral views on the first postoperative day.

**Figure 4:**
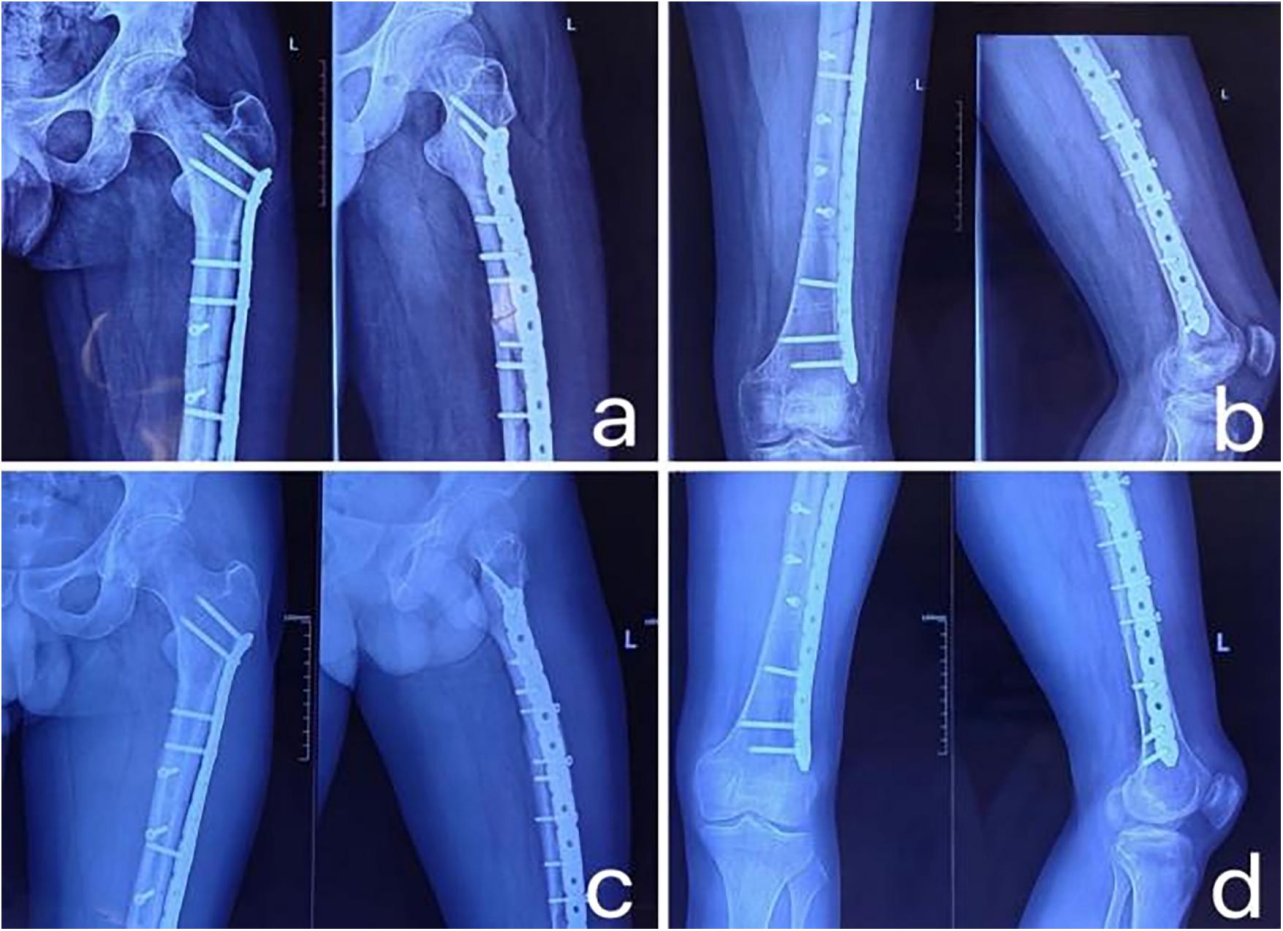
**(a,b)** x-ray images of the femoral shaft in anteroposterior and lateral views taken at the third-month follow-up, showing good fracture alignment with callus formation; **(c,d)** x-ray images of the femoral shaft in anteroposterior and lateral views taken at the one-year follow-up, where the fracture line has completely disappeared, meeting the clinical criteria for fracture healing.

**Figure 5:**
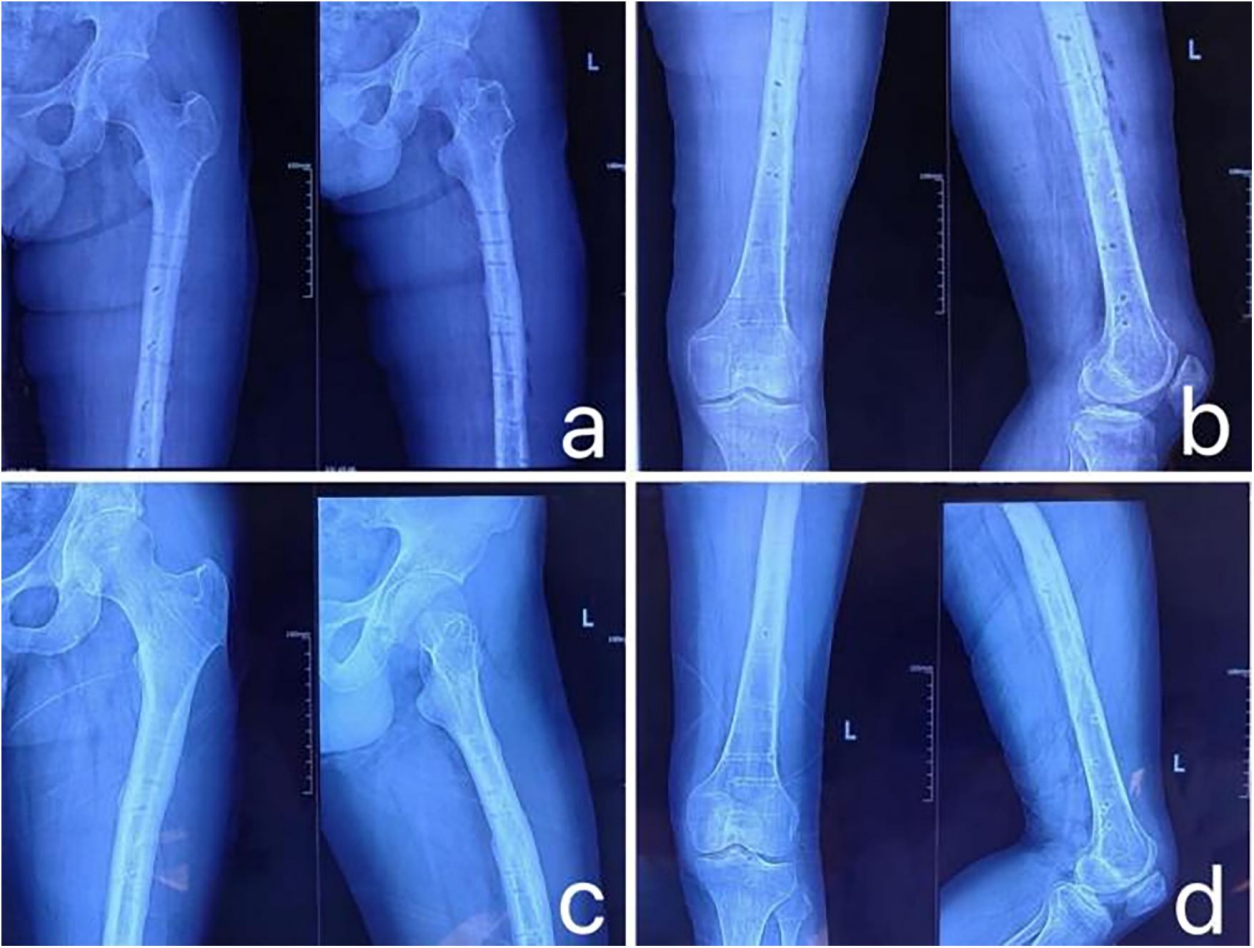
**(a,b)** x-ray images of the femoral shaft in anteroposterior and lateral views taken one year post-surgery after the removal of the internal fixation device; **(c,d)** x-ray images of the femoral shaft in anteroposterior and lateral views taken six months after the removal of the internal fixation device, showing that some screw holes have healed significantly and the fracture site has recovered well.

3D Printing Group: Through an anterior-lateral incision on the femoral shaft, the space between the rectus femoris and vastus lateralis was entered to expose the fracture site, while preserving the periosteum and blood supply to the bone fragments as much as possible. After cleaning the soft tissues at the fracture ends, fracture reduction was performed according to the pre-made 3D physical model. Manual traction and bone holders were used to assist with reduction, and once the fracture position was satisfactory, temporary fixation was done with Kirschner wires. Intraoperatively, fluoroscopy was used to confirm good fracture reduction, and a personalized steel plate was selected. The plate was accurately fixed at the pre-designed bony landmarks, using the model as a guide. Subsequently, a pre-designed steel plate with specific screw holes was used for fixation. To ensure the accuracy of reduction and fixation, multi-angle fluoroscopy was employed during the procedure to check the fracture reduction, and directional motion testing was performed to assess the stability of the fracture fragments. A typical case of the 3D Printing Group is shown in Figures 3–5.

### Postoperative management

All patients receive antibiotic treatment postoperatively to prevent infection, along with routine pain management and anticoagulation therapy. Once the patient is awake from anesthesia, ankle pump exercises and quadriceps isometric contraction exercises can begin. On the first postoperative day, after rechecking the anteroposterior and lateral x-rays of the femoral shaft to confirm no abnormalities, non-weight-bearing flexion and extension exercises of the affected knee and hip joints can be performed. The patient can use crutches for non-weight-bearing walking training and will undergo regular x-ray checks as planned to monitor the stability of the internal fixation and the healing of the fracture. Between 8 and 12 weeks post-surgery, patients were gradually allowed to begin partial weight-bearing exercises. Based on the results of radiographic examinations during follow-up, the weight-bearing intensity was gradually increased. The criteria for assessing fracture healing include: no significant swelling in the affected limb, no localized tenderness or percussion pain at the fracture site, and no abnormal mobility. The patient should be able to bear weight without significant pain, and the anteroposterior and lateral x-rays should show at least three blurred and continuous cortical fracture lines. The follow-up period should be no less than 12 months or until fracture healing occurs.

### Efficacy evaluation criteria

Compare the general information of the two groups of patients, including age, gender, BMI, injury mechanism, and fracture classification. Record perioperative-related indicators in detail, including 3D printing model time, custom metal plate time, surgical time, intraoperative fluoroscopy times, intraoperative blood loss, time from injury to surgery, total hospital stay, and the occurrence of complications. On the first postoperative day, x-ray imaging was used to assess the fracture reduction. Within the first three months post-surgery, radiographic checks were performed monthly to monitor the progress of fracture healing. Afterward, patients underwent radiographic examinations every three months until the fracture was fully healed, and the specific time required for fracture healing was recorded. Three months after the fracture surgery and at the final follow-up, the VAS score was used to assess the level of pain the patient experiences during daily activities. Knee joint function was evaluated through the knee flexion-extension ROM and the HSS knee score (14). At the same time, hip joint function was assessed using the hip flexion-extension ROM and the Harris hip score. The quality of fracture reduction was assessed through x-rays and CT scans, combined with analysis using the 3D SLICER software to evaluate lateral displacement, angular deformity, and shortening deformity at the final follow-up. Based on CT images, the long axis of the femoral shaft was first identified and drawn, then the perpendicular distance from the fracture fragment's cortical edge farthest from the long axis to the long axis was measured. This distance serves as the quantitative indicator of lateral displacement. Postoperative lateral displacement is often a potential precursor to varus or valgus deformity in the coronal plane and also indicates insufficient cortical contact. Poor cortical contact reduces the construct stiffness, alters stress distribution, and creates an unstable micromotion environment, all of which are unfavorable for fracture healing. Therefore, the measurement of lateral displacement is not only radiographically meaningful but also serves as an important indicator of the biological environment for fracture healing.Fracture healing was evaluated using the modified Radiographic Union Score for Tibia (mRUST) (15). This scoring system is typically used to assess tibial fractures but is also applicable for evaluating fracture healing in long bones such as the femur. The mRUST score mainly evaluates the continuity of cortical bone and the quality of the callus in four different regions (anterior, posterior, medial, and lateral) of the fracture site. Each region is scored based on the quality of the callus and the continuity of the cortical bone, with a range of 1–4 points. The total score ranges from 4 to 16 points, with higher scores indicating better fracture healing. In the radiographic evaluation of fracture reduction quality and healing, each indicator was measured three times, and the average of the three measurements was used in statistical analysis.

### Statistical analysis

Data analysis was performed using SPSS 27.0 statistical software. Categorical data were expressed as frequencies or percentages, and the chi-square test was used for inter-group comparisons. For continuous variables, the data were presented as mean ± standard deviation. Before analyzing continuous data, the Shapiro–Wilk test was used to check if the data followed a normal distribution. For continuous variables that followed a normal distribution, independent samples *t*-tests were used to compare inter-group differences. If the data did not follow a normal distribution, the Mann–Whitney *U*-test was used for non-parametric testing. In these analyses, a *P*-value of <0.05 was considered statistically significant.

### Typical case

The patient is a 24-year-old male who sustained a comminuted fracture of the left femoral shaft due to a motor vehicle collision. He received treatment at our hospital using 3D printing technology combined with a personalized metal plate.

## Results

### Baseline demographic characteristics

A total of 36 patients were included in this study. There were 18 patients in the conventional group and 18 patients in the 3D printing group. There were no statistically significant differences in age, gender, BMI, injury mechanism, and fracture classification between the two groups (all *P* > 0.05), indicating comparability (Table 1).

**Table 1:** Baseline demographic characteristics of patients.

Characteristics	Conventional group (*n* = 18)	3D printing group (*n* = 18)	*P* value
Age (years)	36.89 ± 7.44	34.61 ± 7.93	0.381
Gender (*n*)			0.725
Male	11	13	
Female	7	5	
BMI (kg/m²)	24.25 ± 2.54	24.87 ± 2.83	0.497
Injury mechanism (*n*)			0.804
Traffic accident	10	12	
Fall injury	3	2	
Other injuries	5	4	
Winquist-Hansen (*n*)			0.827
Type II	4	3	
Type III	8	10	
Type IV	6	5	

### Comparison of perioperative data

The average time for creating the fracture model in the 3D printing group was 17.06 ± 2.82 h, while the average time for customizing the metal plate was 33.72 ± 4.04 h. The average surgical time for the 3D printing group was 68.83 ± 10.23 min, which was shorter than the 91.72 ± 13.27 min in the conventional group. The 3D printing group also had fewer intraoperative fluoroscopy times (4.39 ± 1.20 times) compared to the conventional group (21.22 ± 2.58 times), with both differences being statistically significant (all *P* < 0.001). The intraoperative blood loss in the 3D printing group was 213.89 ± 40.02 mL, which was less than the 239.17 ± 47.32 mL in the conventional group. The time from injury to surgery was 5.28 ± 0.96 days in the 3D printing group, which was longer than the 4.83 ± 0.71 days in the conventional group. The hospital stay for the 3D printing group was 9.11 ± 1.18 days, which was longer than the 8.83 ± 1.29 days in the conventional group, but the differences were not statistically significant (all *P* > 0.05) (Table 2).

**Table 2:** Comparison of perioperative data.

Parameter	Conventional group (*n* = 18)	3D printing group (*n* = 18)	*P* value
3D Printing Model Time (h)	–	17.06 ± 2.82	–
Customizing metal plate time (h)	–	33.72 ± 4.04	–
Surgical time (min)	91.72 ± 13.27	68.83 ± 10.23	<0.001
Intraoperative fluoroscopy (times)	21.22 ± 2.58	4.39 ± 1.20	<0.001
Intraoperative blood loss (mL)	213.89 ± 40.02	239.17 ± 47.32	0.252
Time from injury to surgery (d)	4.83 ± 0.71	5.28 ± 0.96	0.192
Hospital stay (d)	8.83 ± 1.29	9.11 ± 1.18	0.506

### Comparison of follow-up data

The 3D printing group had shorter times to partial weight-bearing, full weight-bearing, and fracture healing compared to the conventional group (*P* < 0.001, *P* < 0.001, *P* = 0.009). At the three-month and final follow-up, the 3D printing group showed significantly better knee flexion-extension ROM, HSS score, hip flexion-extension ROM, and Harris hip score compared to the conventional group (all *P* < 0.001). There were no statistically significant differences in VAS scores between the two groups (*P* > 0.05). In the conventional group, 1 case of fat liquefaction, 1 case of calf intermuscular thrombosis, and 1 case of nonunion were observed. In the 3D printing group, 1 case of fat liquefaction occurred. There was no statistically significant difference in the incidence of complications between the two groups (*P* = 0.596). For the patient with nonunion, fracture healing was successfully achieved after intramedullary nail dynamization at 12 months post-surgery, and all other adverse reactions resolved within 1 week after treatment. The follow-up time for both groups was at least 12 months (Table 3).

**Table 3:** Comparison of follow-up data.

Parameter	Conventional group (*n* = 18)	3D printing group (*n* = 18)	*P* value
Time to partial weight-bearing (d)	92.22 ± 9.50	81.89 ± 6.93	<0.001
Time to full weight-bearing (d)	136.83 ± 12.07	116.72 ± 11.26	<0.001
Fracture healing time (w)	22.33 ± 2.95	20.06 ± 1.83	0.009
Follow-up Time (m)	13.22 ± 1.00	13.17 ± 1.04	0.864
Knee flexion-extension ROM (°)			
3 months postoperative	104.17 ± 7.43	116.11 ± 6.76	<0.001
Final follow-up	125.28 ± 6.96	134.72 ± 5.81	<0.001
HSS (score)			
3 months postoperative	64.39 ± 8.51	75.61 ± 7.69	<0.001
Final follow-up	80.89 ± 6.39	89.28 ± 4.98	<0.001
Hip flexion-extension ROM (°)			
3 months postoperative	85.39 ± 10.16	105.17 ± 8.22	<0.001
Final follow-up	116.83 ± 8.81	130.06 ± 7.84	<0.001
Harris (score)			
3 months postoperative	60.89 ± 9.02	73.11 ± 8.48	<0.001
Final follow-up	72.89 ± 7.72	85.44 ± 7.02	<0.001
VAS (score)			
3 months postoperative	2.39 ± 0.78	2.56 ± 0.86	0.584
Final follow-up	0.94 ± 0.73	0.72 ± 0.67	0.406
Complication, *n* (%)			0.596
Yes	3 (16.67)	1 (5.56)	
No	15 (83.33)	17 (94.44)	

### Fracture reduction quality and healing assessment

At the final follow-up, fracture reduction quality and healing were assessed for both groups. In terms of lateral displacement, the 3D printing group had an average of 1.15 ± 0.52 mm, which was lower than the conventional group's 2.58 ± 1.12 mm (*P* < 0.001). For angular deformity, the 3D printing group had an average of 1.33 ± 0.69°, which was lower than the conventional group's 3.22 ± 0.73° (*P* < 0.001). Regarding shortening deformity, the 3D printing group had an average of 1.88 ± 0.73 mm, which was also superior to the conventional group's 2.93 ± 0.96 mm (*P* < 0.001). Additionally, the 3D printing group showed better healing outcomes with an average mRUST score of 15.06 ± 0.87, higher than the conventional group's 13.39 ± 1.54 (*P* < 0.001) (Table 4).

**Table 4:** Fracture reduction quality and healing assessment.

Parameter	Conventional group (*n* = 18)	3D printing group (*n* = 18)	*P* value
Lateral displacement (mm)	2.58 ± 1.12	1.15 ± 0.52	<0.001
Angular deformity (°)	3.22 ± 0.73	1.33 ± 0.69	<0.001
Shortening deformity (mm)	2.93 ± 0.96	1.88 ± 0.73	<0.001
mRUST (score)	13.39 ± 1.54	15.06 ± 0.87	<0.001

## Discussion

For long segment comminuted fractures, misalignment and displacement at the fracture site may lead to limb shortening, resulting in leg length discrepancy (16, 17). This discrepancy can cause gait abnormalities, uneven joint loading, and decreased mobility (18, 19). Open reduction surgery can significantly improve fracture reduction quality and effectively prevent common complications. In treating such complex fractures, open reduction is usually combined with plate fixation. Although locking plates provide stable fixation, they may face limitations such as stress shielding, stress concentration, and fixed hole spacing, which can affect fracture healing and long-term stability (20, 21).

To address this issue, our research team utilized 3D printing technology to design customized metal plates. This technology has previously been applied in other fields. Jo et al. (22) compared the effectiveness of traditional surgical methods and the use of 3D printing technology to create customized steel plates for treating pelvic fractures. They found that the use of customized plates allowed for more accurate fracture reduction in the virtual reduction model. Liang et al. (9) applied 3D printing technology in combination with personalized steel plates for the treatment of complex distal intra-articular trimalleolar fractures. They discovered that the personalized steel plate group significantly reduced surgical time, fracture reduction and fixation time, intraoperative fluoroscopy frequency, and incision length, while showing better ankle function scores during follow-up. Assink et al. (23) simulated six cases of tibial plateau fractures using three preserved cadavers, designing custom plates with drilling guides using 3D printing technology to facilitate accurate fracture reduction, correct tibial alignment, and precise screw placement. In the treatment of diaphyseal fractures, Hu et al. (24) utilized 3D printing-assisted minimally invasive percutaneous plate osteosynthesis to manage 42 cases of complex mid-to-proximal humeral shaft fractures, all of which achieved successful bone union. However, studies combining 3D printing technology with patient-specific plates for the treatment of diaphyseal fractures remain relatively limited.

Therefore, this study innovatively applies 3D printing technology in the treatment of long segment comminuted femoral shaft fractures. This technology has shown significant advantages in treating long segment comminuted fractures. Firstly, it overcomes the limitations of traditional plates in dealing with long fracture lines, ensuring a more uniform distribution of stress at the fracture site, while achieving a more precise fit, which in turn minimizes soft tissue damage (25). Secondly, the application of physical models allows surgeons to pre-determine the shape, size, and optimal placement of the plate based on the patient's anatomical features. During surgery, surgeons can also flexibly adjust the direction and position of screw holes according to the actual situation. Lastly, through preoperative virtual surgery design and physical model creation, not only was the surgical plan optimized, but a clear anatomical view was also provided. This personalized treatment approach ensures a perfect fit between the plate and the bone, reducing the likelihood of postoperative displacement. At the same time, it effectively reduces surgical time, intraoperative blood loss, and fluoroscopy frequency, promoting early recovery for the patient and significantly improving the fracture healing process (26, 27).

In this study, due to the need for model creation and custom plate fabrication in the 3D printing group, the time from injury to surgery and the total hospital stay were longer than in the conventional group. However, these differences were not statistically significant (all *P* > 0.05). Notably, the 3D printing group had significantly shorter surgical time and fewer intraoperative fluoroscopy sessions compared to the conventional group (*P* < 0.001), while intraoperative blood loss was slightly higher in the 3D printing group (*P* = 0.252). These results suggest that, compared to traditional limited open reduction and intramedullary nailing, 3D printing technology can effectively shorten surgical time and reduce radiation exposure to patients without significantly increasing intraoperative blood loss, demonstrating its clinical potential. These advantages may be closely related to the different surgical approaches and the use of patient-specific customized plates. The 3D printing group adopted an open approach, which may have simplified the fracture reduction process, while the customized plates, by precisely matching the fracture characteristics, reduced the time required for plate adjustments. The 3D printing group also had shorter times to start weight-bearing, complete weight-bearing, and fracture healing compared to the conventional group (*P* < 0.001, *P* < 0.001, *P* = 0.009). Three months postoperatively and at the final follow-up, the 3D printing group showed significantly better knee flexion-extension ROM, HSS score, hip flexion-extension ROM, and Harris score than the conventional group (all *P* < 0.001). Although the incision for open reduction and internal fixation with a plate is longer, causing greater trauma to the body, there were no statistically significant differences in VAS scores between the two groups at any time point (*P* > 0.05). The results suggest that, despite the relatively larger surgical trauma in the 3D printing group, the significant improvement in postoperative function may, to some extent, offset the negative impact of pain. This effect is likely closely related to the precise fitting of the custom plates, which optimized fracture alignment, accelerated bone healing, and provided patients with the opportunity for earlier functional recovery, thereby promoting the postoperative rehabilitation process. At the final follow-up, the 3D printing group showed better outcomes in terms of lateral displacement, angulation deformity, shortening deformity, and mRUST score at the fracture site (all *P* < 0.001). These results indicate that 3D printing technology may have advantages in promoting fracture healing and recovery.Although the radiological results show statistically significant differences, the absolute differences between the two groups are small, so the actual impact on patient prognosis may be limited. Statistically, 3D printing technology appears to improve deformity parameters during the fracture healing process to some extent. However, from a clinical perspective, the clinical significance of these improvements may be modest, as long-term functional recovery and quality of life for patients are influenced by multiple factors, not just the specific values of deformity parameters.

In this study, there were significant differences in the surgical techniques used between the two groups in terms of anatomical location and surgical approach. The conventional group adopted closed reduction and intramedullary nail fixation. However, since the femoral shaft fractures in this study were all characterized by long and comminuted fracture lines, many patients still required a longer incision for assisted reduction. Compared to traditional closed reduction, this surgical approach not only increased intraoperative blood loss but also caused greater trauma, particularly to the vastus lateralis and fascia, with a higher risk of postoperative adhesion. Moreover, due to the long and comminuted nature of the fractures, the conventional group was fixed with only two screws at both the proximal and distal ends, which prevented early weight-bearing after surgery, thereby affecting the patients' rehabilitation process. Therefore, the functional scores in the intramedullary nail group were lower than expected and not entirely consistent with previous literature, primarily because the study focused on complex femoral shaft fractures (long-segment, highly comminuted), whose unique fracture characteristics may have affected the efficacy of conventional intramedullary nailing.In contrast, the 3D printing group, with precise fracture fixation, was able to bear weight early, which facilitated functional recovery. These factors may be one of the reasons for the lower knee joint range of motion in the conventional group compared to the 3D printing group. The results of this study do not undermine the value of intramedullary nailing itself, but rather highlight that its efficacy may vary across different fracture types. In cases of extremely complex comminuted femoral shaft fractures, the inherent technical characteristics of the procedure may encounter challenges during implementation, potentially affecting the ultimate functional outcome.

We acknowledge that in this study, the choice of surgical method may have been influenced by fracture complexity. Specifically, for patients with complex fracture patterns or multi-fragmentary fractures, surgeons may have been more likely to select 3D-printed, patient-specific plate fixation. This preferential selection could introduce treatment allocation bias. Although baseline characteristics were carefully matched in this study, fracture complexity remains a potential confounding factor that may affect the assessment of surgical outcomes and, consequently, the direct comparison of clinical and radiographic results between groups.

Although 3D printing technology and personalized plates have shown numerous advantages in the treatment of long-segment comminuted fractures, there are still some limitations in clinical application. First, the use of 3D printing technology relies on high-precision equipment and specialized personnel, resulting in higher costs compared to traditional surgical methods (28). Additionally, the printing and customization process still requires a significant amount of time, which may prolong the patient's hospital stay and potentially impact the overall treatment process. Finally, the time and economic costs of 3D printing technology may become potential limiting factors for its widespread application. The high costs of 3D printing and custom-made plates, along with the long production cycles, may not meet the urgent demands of emergency surgeries. Although this process is relatively time-consuming and costly, individualized plates can significantly improve intraoperative fit and fracture reduction accuracy, reduce intraoperative adjustment time and the risk of complications, and thus offer certain advantages from the perspective of potential clinical benefits.

### Limitations

This study has several limitations. First, this is a retrospective, non-randomized study, and the results should be interpreted as associations observed between different fixation methods within this study cohort rather than evidence of causality. Second, the follow-up period is relatively short, which limits the ability to comprehensively assess long-term efficacy and potential complications. Lastly, due to the small sample size, this study may not fully reflect the broad application of 3D printing technology combined with personalized plates. Therefore, future large-scale, randomized controlled multicenter studies are needed to further validate the effectiveness and safety of 3D printing technology and personalized plates in the treatment of long-segment femoral shaft comminuted fractures. Additionally, more cost-effective 3D printing technologies and materials need to be developed to reduce surgical costs and make this technology accessible to a larger number of patients.

## Conclusion

In this study cohort, 3D-printed-assisted fracture internal fixation demonstrated superior outcomes in both clinical efficacy and radiographic assessment compared with intramedullary nail fixation. These findings suggest that this approach may help improve surgical precision and fracture reduction quality, with the potential to facilitate early functional recovery in patients.

## Data Availability

The original contributions presented in the study are included in the article/Supplementary Material, further inquiries can be directed to the corresponding author.
